# A negative correlation between behavioural and physiological performance under ocean acidification and warming

**DOI:** 10.1038/s41598-018-36747-9

**Published:** 2019-03-12

**Authors:** Taryn D. Laubenstein, Jodie L. Rummer, Mark I. McCormick, Philip L. Munday

**Affiliations:** 1 ARC Centre of Excellence for Coral Reef Studies, James Cook University, Townsville, QLD 4811 Australia; 2 College of Science and Engineering, James Cook University, Townsville, QLD 4811 Australia

## Abstract

Many studies have examined the average effects of ocean acidification and warming on phenotypic traits of reef fishes, finding variable, but often negative effects on behavioural and physiological performance. Yet the presence and nature of a relationship between these traits is unknown. A negative relationship between phenotypic traits could limit individual performance and even the capacity of populations to adapt to climate change. Here, we examined the relationship between behavioural and physiological performance of a juvenile reef fish under elevated CO_2_ and temperature in a full factorial design. Behaviourally, the response to an alarm odour was negatively affected by elevated CO_2_, but not elevated temperature. Physiologically, aerobic scope was significantly diminished under elevated temperature, but not under elevated CO_2_. At the individual level, there was no relationship between behavioural and physiological traits in the control and single-stressor treatments. However, a statistically significant negative relationship was detected between the traits in the combined elevated CO_2_ and temperature treatment. Our results demonstrate that trade-offs in performance between behavioural and physiological traits may only be evident when multiple climate change stressors are considered, and suggest that this negative relationship could limit adaptive potential to climate change.

## Introduction

A major goal of climate change research is to determine whether species will withstand environmental change. A wealth of studies has assessed biological responses to projected future conditions in a variety of taxa, providing an indicator of large-scale trends in response to climate change conditions^1–3^. However, it can be advantageous to look beyond mean responses and examine the variation in performance between individuals within a species or population. Once disregarded and considered to be noise or error between measurements, individual variation is now recognized as a metric worth investigating in its own right^4,5^. In particular, a surge of interest has grown around variation in behavioural and physiological traits for their potential to identify patterns of trait covariation^4,6^, determine the mechanistic underpinnings of these covariations^5,7,8^, and predict their evolutionary implications^9,10^.

The relationship between behavioural and physiological performance could be especially important in the context of climate change. Examining this type of variation can highlight the individuals that are best suited to survival in future climate conditions, as well as reveal correlations between types of performance that can either help or hinder survival at the individual level^11^. Furthermore, correlations between traits could have implications for the potential of organisms to adapt to climate change^11,12^. In particular, if the behavioural and physiological traits of interest are heritable, then correlations between them could either accelerate or decelerate adaptive evolution^10,13^. If the traits are negatively correlated, selection on one would diminish the other, decreasing the rate of adaptation, and vice versa. Thus, identifying correlations among key traits is an important step in predicting species persistence in the face of climate change^11,12,14^. Importantly, environmental stressors have been shown to alter the relationship between behavioural and physiological traits, either amplifying or masking significant correlations^15^. Therefore, to improve our understanding of the effects of climate change on fish, it will be necessary to evaluate these relationships not only under current-day conditions, but also under different climate change-relevant scenarios.

Ocean acidification and warming are two of the primary environmental stressors in marine ecosystems^16,17^. As both are driven by increasing carbon dioxide emissions, they will likely increase in tandem, forcing marine organisms to contend with both stressors simultaneously^18,19^. The majority of climate change studies on marine organisms have focused on single stressors, although there has been an increasing recognition of the importance of a multi-stressor approach^2,20^. Multi-stressor experiments more accurately capture likely future scenarios, and can reveal unexpected interactions between stressors. For instance, ocean acidification and warming have been shown to interact both synergistically and antagonistically on the responses of marine organisms^20,21^. Thus, multi-stressor experiments are crucial, as single-stressor studies could lead to inaccurate predictions about organismal responses to future climate conditions^20,22^.

Reef fishes can be sensitive to the effects of both elevated CO_2_ and temperature. Elevated CO_2_ levels projected for the end of the century have been documented to impact a range of behavioural traits in reef fishes such as lateralization, activity, homing ability, learning, anxiety, and olfactory and auditory discrimination (reviewed by Clements & Hunt^23^ and Nagelkerken & Munday^24^). These behavioural changes can make fish more vulnerable to predation and have been linked to higher mortality rates^25–27^. Elevated CO_2_ has also been documented to affect physiological traits, such as aerobic scope^28,29^, escape performance^27^, and reproduction^30,31^ in some reef fishes, but the physiological effects of CO_2_ are generally more variable across species than behavioural effects^32,33^. By contrast, temperature is well known to impact fish physiological performance through its effect on rates of biochemical reactions^34^. Elevated temperatures predicted for the end of the century have been shown to alter aerobic scope^35–37^, body size^38^, reproductive output^39,40^, and swimming ability^41^ in reef fishes. Behaviourally, both boldness and activity levels have been shown to change in response to temperature increases^42–44^.

In this study, we investigated the effects of elevated CO_2_ and temperature on behavioural and physiological performance of juvenile spiny chromis, *Acanthochromis polyacanthus*. We used a full factorial design which consisted of a current-day ambient CO_2_ level (~500 µatm) and average summer temperature for the study location (29° C), crossed with elevated CO_2_ (~1000 µatm) and temperature (32° C) based on projections for the end of the century under RCP 8.5^17,45^. The behavioural trait we measured was the percent reduction in feeding strikes after exposure to damage-released olfactory cues for a conspecific (i.e., alarm odours). Conspecifics display an innate aversion to these reliable indicators of predation risk, typically reducing activity and seeking shelter^46^. However, previous studies have found that when fishes are reared under elevated CO_2_ levels, they no longer reduce their activity or stop feeding in the presence of these odours^47,48^. The inability to use alarm odours under elevated CO_2_ conditions makes prey more vulnerable to predators and can lead to higher mortality at key life-history stages^47,49^. The physiological trait we determined was aerobic scope. Aerobic scope refers to the total capacity for aerobic activity available to an organism, after accounting for basic maintenance^34,50^, and is calculated as the difference between the maximal and resting oxygen uptake rates of an organism. Aerobic activities such as growth, reproduction, and swimming are essential life-history processes; therefore, a reduction in aerobic scope could reduce individual performance and fitness^1,51,52^. Both elevated CO_2_ and elevated temperature have been predicted to decrease aerobic scope in fishes^1,53^. However, when tested, elevated temperature often decreases aerobic scope in coral reef fishes^35,36^, while elevated CO_2_ has had more variable effects^32,33^.

By tracking the responses of individual fish when tested for their behavioural and physiological performance, we were able to determine the relationship between these traits at the individual level. We then compared this relationship between all four of the treatment groups to determine how the relationship between these traits might change under different environmental stressors, and ultimately, how adaptation to climate change conditions might be either facilitated or hindered by this relationship.

## Results

### Behaviour

**Figure 1:**
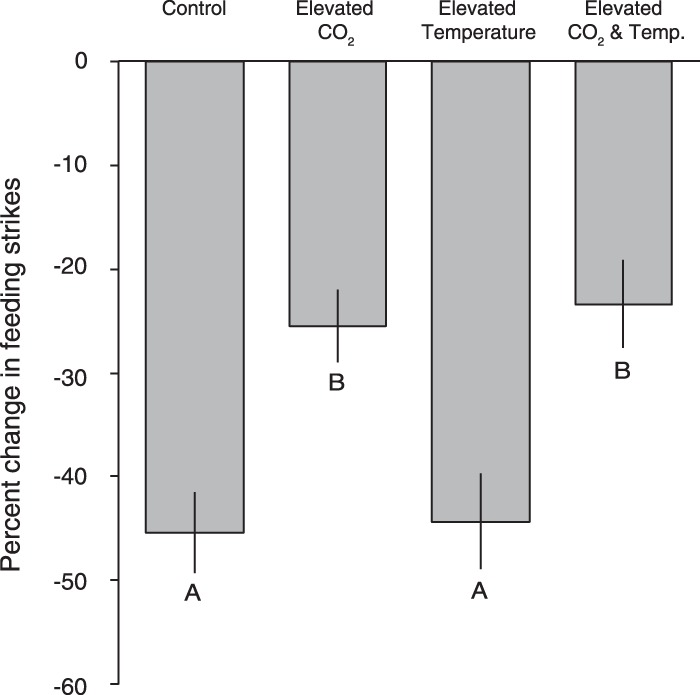
The effect of elevated CO_2_ and temperature on percent change in feeding strikes in juvenile spiny chromis damselfish following the addition of an alarm odour. Values are means ± SE. Letters represent Tukey’s HSD groups. N = 38–47 per treatment.

There was a highly significant effect of CO_2_ treatment on percent reduction in feeding strikes following the addition of the alarm odour (*t*_160_ = −3.69, P < 0.001, Fig. 1). Fish from the two current-day control CO_2_ treatments exhibited a 43.3% greater reduction in feeding strikes than fish from the two elevated CO_2_ treatments. Temperature treatment did not affect the reduction in feeding strikes (*t*_160_ = −0.03, P = 0.98). There was no interaction between CO_2_ and temperature on percent reduction in feeding strikes (*t*_160_ = −0.23, P = 0.82).

### Physiology

**Figure 2:**
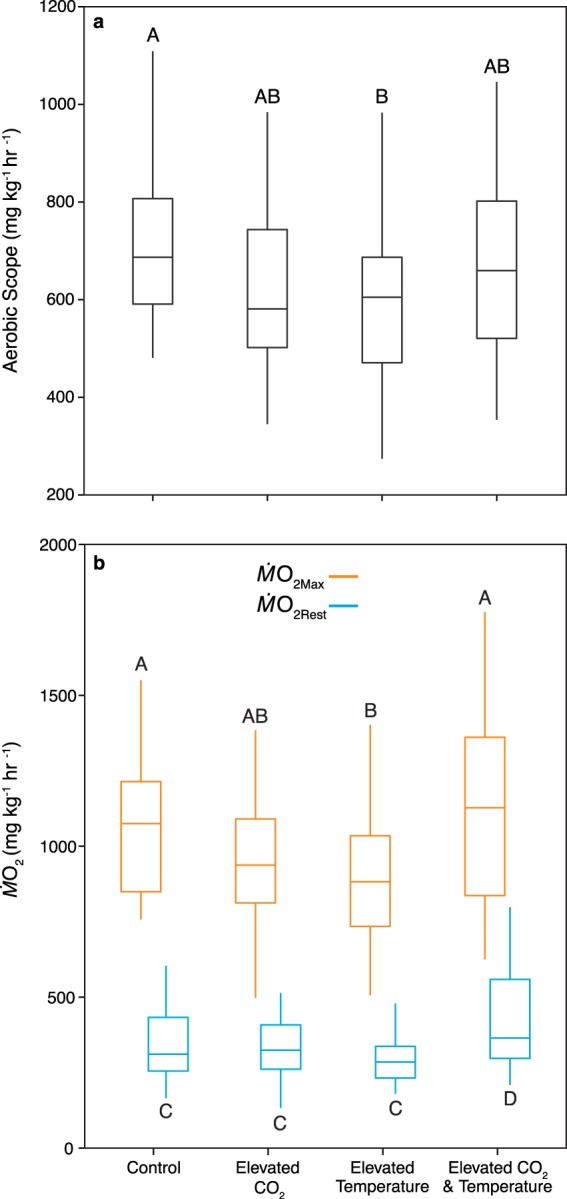
The effect of elevated CO_2_ and temperature treatments on resting and maximal oxygen uptake rates and aerobic scope in juvenile spiny chromis damselfish. Boxplots show median and inter-quartile range for (**A**) absolute aerobic scope (*Ṁ*O_2Max_ - *Ṁ*O_2Rest_); and (**B**) resting (*Ṁ*O_2Rest_; blue boxes) and maximal oxygen uptake rates (*Ṁ*O_2Max_; orange boxes). Letters represent Tukey’s HSD groups. N = 32–38 per treatment.

There was a significant interaction between CO_2_ and temperature treatments on aerobic scope (*t*_134_ = 2.15, P = 0.03; Fig. 2A). Tukey’s post-hoc tests revealed that there was a significant difference in aerobic scope between the control and elevated temperature treatments held at control CO_2_ (z = −4.03, P = 0.001), but this was not the case when CO_2_ was elevated (pairwise interactions, P > 0.05). Despite the significant interaction, there was a strong effect of temperature evident in the data, which was supported by a significant main effect of temperature (*t*_134_ = −3.73, P < 0.001). Fish from the two elevated temperature treatments exhibited a 20% lower aerobic scope than fish from the two control temperature treatments. There was no main effect of CO_2_ treatment on aerobic scope (*t*_134_ = −1.43, P = 0.15), while mass had a significant effect on aerobic scope (*t*_134_ = −5.19, P < 0.001).

There was a significant interaction between CO_2_ treatment and temperature treatment on *Ṁ*O_2Max_ (*t*_140_ = 3.47, P < 0.001; Fig. 2B). Tukey’s post-hoc tests revealed that juveniles reared in the current-day control and in the combined elevated CO_2_ and temperature treatments had a significantly higher *Ṁ*O_2Max_ than juveniles from the elevated temperature treatment (control: *z* = 3.58, P = 0.002; elevated CO_2_ and temperature: *z* = 3.61, P = 0.002), while juveniles from the elevated CO_2_ treatment had a *Ṁ*O_2Max_ which was not significantly different from the other three treatments (pairwise interactions, P > 0.05). As was the case with aerobic scope, there a significant main effect of temperature treatment on *Ṁ*O_2Max_ (*t*_140_ = −3.58, P < 0.001). Fish from the two elevated temperature treatments had an 18.2% lower *Ṁ*O_2Max_ than fish from the two control temperature treatments. There was no main effect of CO_2_ treatment on *Ṁ*O_2Max_ (*t*_140_ = −1.29, P = 0.20) and mass had a highly significant effect on *Ṁ*O_2Max_ (*t*_140_ = −3.32, P = 0.001).

There was a significant interaction between CO_2_ treatment and temperature treatment on *Ṁ*O_2Rest_ (*t*_136_ = 3.45, P < 0.001; Fig. 2B). Post-hoc Tukey’s tests revealed that juveniles reared in the current-day control, elevated temperature, and elevated CO_2_ treatments all had similar values for *Ṁ*O_2Rest_ (pairwise interactions, P > 0.05), whereas juveniles reared in the elevated CO_2_ and temperature treatment had a significantly higher *Ṁ*O_2Rest_ (pairwise interactions, P < 0.05). Neither CO_2_ treatment nor mass had a significant effect on *Ṁ*O_2Rest_ (*t*_136_ = −0.40, P = 0.69 and *t*_136_ = 0.05, P = 0.96, respectively).

### Relationship between Behavioural and Physiological Performance

**Figure 3:**
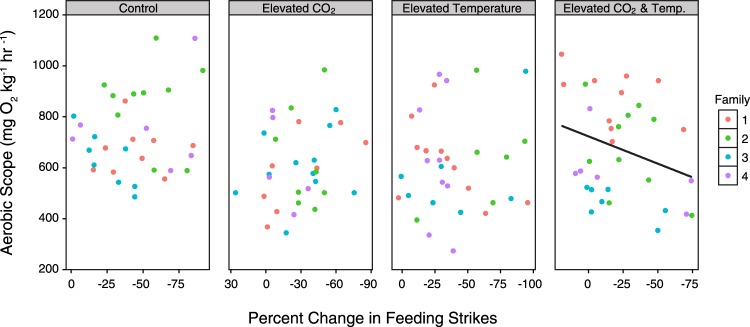
The relationship between percent change in feeding strikes and aerobic scope. Panels represent different treatments, and colours represent different family groups. Trend lines are shown as derived from linear mixed effect models. The relationship is statistically significant for the elevated CO_2_ and temperature treatment.

**Figure 4:**
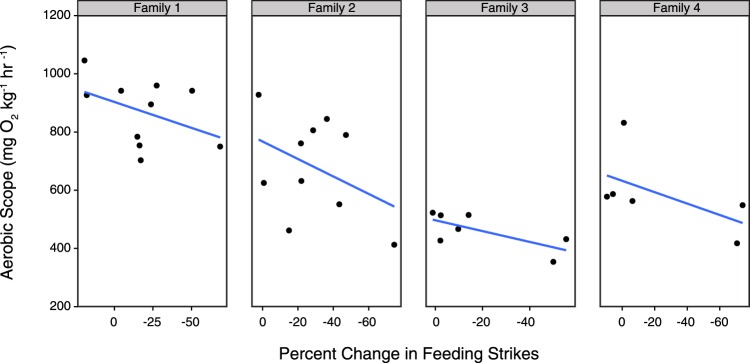
The relationship between percent change in feeding strikes and aerobic scope for the elevated CO_2_ and temperature treatment. Panels represent different family groups. Trend lines are shown as derived from linear models.

There was no significant relationship between percent reduction in feeding strikes and aerobic scope in the juveniles reared in the current-day control (*t*_27_ = −0.29, P = 0.77, Fig. 3), elevated CO_2_ (*t*_26_ = 1.72, P = 0.10), or elevated temperature (*t*_27_ = 0.22, P = 0.83) treatments. However, there was a significant negative relationship between percent reduction in feeding strikes and aerobic scope in juveniles reared at elevated CO_2_ and temperature (*t*_27_ = −3.09, P = 0.005). This relationship was consistent across all family groups (ANOVA of LM, Family x Reduction in Feeding Strikes interaction, P > 0.05, Fig. 4).

## Discussion

Our study found a negative relationship between changes in feeding strikes in response to alarm odour and aerobic scope, but only when fish were reared in elevated CO_2_ and temperature conditions. Our results indicate that when exposed to two climate change stressors, there are no winner and loser individuals– rather, each individual seems constrained along a maximal performance ridge^14^, such that an individual could maintain a relatively high aerobic scope, or an ecologically appropriate response to alarm cue, but not both. Furthermore, the relationship was consistent across family groups, suggesting that certain families do not hold a distinct advantage over others in dealing with this limitation. This negative correlation could have implications for the adaptive potential of this species. Selection for improved performance of either trait could decrease performance in the other, slowing adaptation. Given the rapid pace at which the global environment is changing, any factor that slows or limits selection for improved performance could have serious implications for individual performance and ultimately, population success.

We detected a significant correlation between traits only in the multi-stressor treatment. This result aligns well with theory that environmental stress can act as a revealing or amplifying factor on correlations between behaviour and physiology^15^. The mechanism for this pattern is not known, but may be explained by differing sensitivities to the stressors between individuals, which can increase the intraspecific phenotypic variation in the traits^54,55^. Additionally, the increased demands on performance that are imposed by stressors can emphasize the importance of certain traits, making links between behaviour and physiology more evident under stressful conditions^15^. Indeed, since we observed behavioural performance to be most affected by elevated CO_2_ and physiological performance to be most affected by elevated temperature, it is intuitive that the presence of both stressors might be necessary to observe this relationship. Unfortunately, ocean waters will likely become both warmer and more acidic in the future, meaning that future conditions may elicit this negative correlation between behavioural and physiological performance.

This study investigated phenotypic, not genotypic correlations between traits. While phenotypic correlations are often indicative of genetic correlations^56^, we did not specifically test the genetic basis of the phenotypic traits considered here. Correlations between phenotypic traits might enhance or hinder selection, but ultimately adaptation can only be influenced by genetic covariation between traits. This means that the observed phenotypic variation must be heritable for adaptation to occur. Recent studies demonstrate that aerobic scope has high heritability under elevated temperatures^57^, and response to alarm odour is heritable, at least under acute exposure to elevated CO_2_^58,59^. These studies suggest that these traits have a significant amount of additive genetic variation, indicating that the correlations we have detected could indeed affect adaptation.

Our behavioural performance trials demonstrated that juveniles reared under elevated CO_2_ conditions did not reduce their feeding strikes after the addition of an alarm odour to the same extent as juveniles reared under current-day control conditions. This is consistent with previous studies that have shown elevated CO_2_ to negatively impact a range of behavioural and sensory traits in reef fishes^23,24^. A likely mechanism underpinning the behavioural changes we observed is a disruption to neurotransmitter receptor function. Fish have robust acid-base regulatory systems that help them to maintain a stable internal pH^60^. However, the changed concentration of acid-base relevant ions that is required to maintain internal pH under elevated CO_2_ conditions can interfere with the function of GABA_A_ neurotransmitter receptors, resulting in altered behaviour and impaired olfactory responses^61,62^. It is important to note that our behavioural assay does not differentiate between fish with impaired olfactory preferences versus those with increased activity and boldness; rather, the assay encompasses both responses in one ecologically-relevant test. Because ocean acidification has been shown to alter a broad range of cognitive functions in marine fishes, it is likely that it affects central neural processing rather than individual behaviours or sensory systems^60,61^. Thus, changes to both boldness and olfactory preference likely stem from the same disruption to GABA_A_ functioning, allowing them to be assessed simultaneously in our assay. Through this method, we were able to portray a real-life scenario involving both foraging and anti-predator response in which a fish must respond appropriately to an alarm odour.

In our physiological performance trials, we documented that the aerobic scope of juvenile *A*. *polyacanthus* decreased under elevated temperature. This negative effect of elevated temperature on aerobic performance has been previously shown in a range of coral reef fishes^35–37^, although the mechanism by which temperature affects aerobic performance in ectotherms is still not fully understood^63^. There was also a non-significant trend toward decreased aerobic scope in fish maintained under elevated CO_2_ conditions. Elevated CO_2_ has been shown to have mixed effects on aerobic scope in a range of marine fishes, with elevated temperature typically having a greater effect than elevated CO_2_^32,33^, which aligns well with our results. However, our results contrast with a 38% increase in aerobic scope that was observed in adult *A*. *polyacanthus* exposed to similar CO_2_ levels^29^. We hypothesize that the differing results primarily stem from the different life-stages of the tested fish (i.e., juvenile vs. sexually-mature adults). It has been suggested that early life stages of fish are more sensitive to changes in pH due to their high surface area-to-volume ratio^64,65^, which could explain this discrepancy. Our results further underscore the importance of considering life stage when determining species responses to elevated CO_2_.

This study indicates that there is a negative correlation between behavioural and physiological performance in juvenile damselfish exposed to elevated CO_2_ and temperature. This relationship could reduce individual performance maxima, as well as limit the potential for adaptive evolution in the population. However, it is important to note that the extent to which this relationship is detrimental will depend upon other factors, such as food availability and predator abundance, and the relative benefits of a higher aerobic scope versus stronger anti-predator response in these environments. Juvenile coral reef fishes are generally considered to live in a high-risk environment, as up to 55% of settlement-stage juveniles are estimated to be consumed within days of reaching a reef^66^, suggesting that effective anti-predator responses would be under strong selection. Similarly, a high aerobic scope is thought to be beneficial when food is abundant, but less helpful when food is scarce^67^, and thus its importance will depend upon food availability. Still, in reef fishes there is evidence for heritability of phenotypic variation in physiological and behavioural traits under elevated CO_2_ and temperature^57–59^, so it is not unreasonable to expect that adaptation can and will act on these traits under future climate conditions.

While we saw a clear correlation in these traits under elevated CO_2_ and temperature conditions, the proximal cause for the relationship remains unknown. Linkages among metabolic traits and behavioural traits have been well explored^7,8,68^, but proximal causes are generally more difficult to identify. For instance, the observed negative correlation might be caused by genetic linkages between traits, but could also be explained by a shared hormonal feature^69^, or a trade-off in energetics between internal pH regulation and thermal tolerance^70^. This work thus represents an important first step in identifying this correlation, and opens an avenue for future research to identify the mechanistic basis of this relationship.

## Materials and Methods

### Study species, brood-stock collection and maintenance

The spiny chromis, *Acanthochromis polyacanthus*, is a tropical damselfish from the western Pacific Ocean. The species has direct development (i.e. eggs hatch into juveniles), and both parents care for the eggs and offspring for up to 45 days post-hatch^71^. Without a pelagic larval phase, the species is easily bred in captivity. Due to its wide geographic distribution, abundance, and amenability to laboratory experimentation, *A*. *polyacanthus* has become a model species for studying the effects of climate change on coral reef fishes (e.g.^39,48^).

Adult *A*. *polyacanthus* were collected using hand nets from the Bramble Reef area (site 1: 18°22′S, 146°40′E; site 2: 18°25′S, 146°40′E) on the Great Barrier Reef in July 2015. Fish were transported to James Cook University (Townsville, Australia), where they were sorted into breeding pairs and housed in 60 L aquaria. Pairs were provided with half a terracotta pot for shelter and as a suitable artificial breeding site. Aquaria were checked daily for the presence of newly laid clutches. Pairs were fed *ad libitum* on commercial fish feed pellets (INVE Aquaculture Nutrition NRD 12/20) once per day outside the breeding season (July–October) and twice per day during the breeding season (November–May). Starting in October, water temperatures were increased at a rate of 0.5° C per week until the summer breeding temperature of 29° C was reached during the first week of November 2015.

Offspring were fed freshly hatched *Artemia* spp. nauplii for the first two days post-hatch (dph), then a combination of *Artemia* spp. nauplii and weaning fish feed (INVE Aquaculture Nutrition Wean-S 200–400  μm) daily for the following three days. They were fed the weaning fish feed from 6–21 dph and then switched to a small pellet fish feed (INVE Aquaculture Nutrition NRD 5/8) at 22 dph.

### Carbonate Chemistry

Water for this experiment was supplied via two 8000 L recirculating aquarium systems. The ambient *p*CO_2_ level (~500 μatm) in one system was used as the current-day control. The other system was dosed with CO_2_ to match the end-of-century projection for surface ocean *p*CO_2_ under RCP 8.5 (~1000 μatm). The *p*CO_2_ level was controlled by an Aqua Medic AT Control System (Aqua Medic, Germany), which dosed CO_2_ into a 3000 L sump connected to the system whenever the pH in the system rose above the set point. An identical 3000 L sump on the current-day control was not dosed with additional CO_2_. Temperature was maintained at a current-day control of 29° C by circulating seawater through a SolarWise heater/chiller (Brisbane, Queensland, Australia) on each system. The equilibrated seawater from each system was then either delivered directly into the aquaria, or passed over Toyesi 2.5-kW inline heaters (Prospect, New South Wales, Australia) to raise the temperature to the elevated treatment of 32° C. Water was delivered into fish aquaria at a rate of 1.5 L min^−1^ in a temperature-controlled room.

The pH_NBS_ and temperature for each system were recorded daily using a pH electrode (SevenGo Pro, Mettler Toledo, Switzerland) and temperature probe (Cormark C26, Norfolk, UK). The pH_T_ was measured weekly with a spectrophotometer following standard operating procedures^72^ using the indicator dye meta/*m*-cresol purple (*m*-cresol purple sodium salt 99%, non-purified, Acros Organic).

Total alkalinity was also estimated weekly by Gran Titration (Metrohm 888 Titrando Titrator Metrohm AG, Switzerland) and using certified reference material from Dr. A.G. Dickson (Scripps Institution of Oceanography). Salinity was measured weekly using a conductivity sensor (HQ15d; Hach, Loveland, CO, USA). All water quality parameters were measured in randomly selected aquaria. The *p*CO_2_ was calculated as a function of pH_T_, temperature, salinity, and total alkalinity in CO_2_SYS using the constants K1 from Mehrbach *et al*.^73^. refit by Dickson & Millero^74^, and KHSO_4_ from Dickson^75^ (Table 1).

**Table 1:** Seawater parameters for the experimental period (21 Jan to 6 May 2016). Values are means ± SD.

Treatment	Temperature (°C)	Salinity (ppt)	pH_T_	Alkalinity (μmol kg^−1^ SW)	*p*CO_2_ (μatm)
Control	29.0 ± 0.2	34.9 ± 1.4	7.94 ± 0.03	2101.4± 129.2	486.7 ± 35.6
Elevated CO_2_	29.0 ± 0.2	35.6 ± 0.5	7.68 ± 0.03	2111.8 ± 100.8	966.8 ± 74.5
Elevated Temperature	31.9 ± 0.2	34.9 ± 1.4	7.89 ± 0.03	2101.4 ± 129.2	545.5 ± 39.9
Elevated CO_2_ & Temp.	31.9 ± 0.2	35.6 ± 0.5	7.64 ± 0.03	2111.8 ± 100.8	1078.1 ± 82.3

### Experimental Design

One clutch of offspring from each of four different parental pairs was used for this experiment. At 1 dph, offspring in each clutch were divided into each of the four CO_2 _X temperature treatment groups: control, elevated CO_2_, elevated temperature, and elevated CO_2_ and temperature (Table 1), representing a 2 × 2 factorial design. Behavioural trials were performed at 60–66 dph, and physiological trials were performed on the same individuals at 62–68 dph, allowing at least one day rest between trials. To track individual fish between trials, immediately following the behavioural trial, individuals were placed into labelled PVC pipes (8 cm diameter, 5 cm length) that were covered at both ends by a thin plastic mesh to allow for flow-through of water, and then placed into treatment tanks. All trials were performed during daylight hours only (09:00–18:00) in the fish’s respective treatment water. Research was carried out under approval of the James Cook University animal ethics committee (permit: A2197) and according to the University’s animal ethics guidelines.

### Behavioural Assay

The percent change in feeding strikes has been widely used to test the behavioural responses of fishes to conspecific alarm cues^44,76–78^, and this method has been used in previous ocean acidification experiments^47,49,79^. Using percent change in feeding strikes rather than the absolute feeding rate can account for differences in activity or feeding rate between individuals. The change in feeding strikes in response to alarm odours was tested using methods similar to those described by Ferrari *et al*.^47^. Trials were conducted in 13 L (36 × 21 × 20 cm) flow-through aquaria. Each tank contained a small shelter made of half a PVC pipe (8 cm diameter) at one end of the tank, and an airstone at the opposite end. Attached to the airstone was an injection tube for adding food and alarm odours to the tank. The end of the tube was located just above the airstone to ensure rapid dispersal of food and alarm odours throughout the tank. The tank was surrounded on three sides with white plastic to visually isolate the fish, and a black plastic curtain with a small flap separated the tank from the observer and other external stimuli.

Single juvenile *A*. *polyacanthus* were placed into tanks filled with their treatment water to habituate overnight (15 h). Each tank received a continuous flow of the relevant treatment water at 0.6 L min^−1^. A maximum of fourteen fish were tested per day. Ten minutes prior to a trial, the flow-through system to the tank was turned off to prevent the alarm odour from washing out of the tank. At this point, the flap in the observation curtain was opened and a camera placed into the opening, to habituate the fish to the camera. The fish was unable to see the observer, but the fish could be viewed on the camera’s screen. Immediately prior to a trial, 20 mL of water were drawn from the injection tube and discarded to remove any stagnant water that might have collected in the tube. A further 60 mL were drawn from the tube for flushing alarm odours into the tank.

Alarm odours were freshly prepared during the first 10 minutes of each trial, as these cues have been shown to lose potency after 20 minutes when kept at room temperature^80^. One juvenile *A*. *polyacanthus* donor was used for each test fish. The donor fish was euthanized with a quick blow to the head. The alarm odour was then prepared by making eight superficial vertical cuts along each side of the body with a scalpel blade. The cuts were rinsed well with 10 mL of seawater, and the cue water was passed through a filter to remove any solid material or scales^47^.

To start a trial, the camera (Canon Powershot G15, Canon Powershot G16, or Canon Powershot GX9) was turned on, and the fish was recorded for five minutes to ensure it did not exhibit any abnormal behaviours. Next, 2.5 mL of *Artemia* solution (containing ~250 individuals per mL) was slowly flushed into the tank with 20 mL of seawater to allow the fish to establish a stable feeding rate. After five minutes, another 2.5 mL of *Artemia* solution was flushed into the tank with 20 mL of seawater. Finally, after another five minutes, 2.5 mL of *Artemia* solution, followed by 10 mL of alarm odour, followed by 20 mL of seawater were flushed into the tank. The trial ended five minutes after the addition of the alarm odour, with each trial totalling 20 minutes. The entire trial was filmed for analysis. Following trials, fish were returned to their rearing tanks. Between 8 and 14 individuals from each of four family groups were tested per treatment, for a grand total of 38–47 fish per treatment.

Videos were analysed by a single researcher (T.L.) to determine the feeding rate of the fish before and after the addition of the alarm odour. Video file names were scrambled so that the viewer was unaware of the treatment. Feeding strikes were counted for four minutes in each time period, allowing 30 seconds after the addition of food or alarm odour to ensure a steady feeding rate, and that the alarm odour had fully permeated the tank. The percent change in feeding strikes after the addition of the alarm odour was then calculated.

### Physiological Assay

To estimate aerobic scope, the maximum (*Ṁ*O_2Max_) and resting (*Ṁ*O_2Rest_) oxygen uptake rates were measured using intermittent flow respirometry, based on standard respirometry methods^81,82^. Fish were starved for 24 hours prior to testing to ensure a post-absorptive state^83^. As *A*. *polyacanthus* is a coral reef fish with a relatively sedentary lifestyle, *Ṁ*O_2Max_ was measured following a chase to exhaustion (3 min.) and a brief (1 min.) air exposure, as this method has been shown to reliably capture *Ṁ*O_2Max_^82^. Following the chase and air exposure, fish were then immediately placed into darkened glass respirometry chambers (38 mL total volume including tubing) that were submerged in aquaria containing the fish’s treatment water. Submersible pumps fitted to each chamber supplied a continuous flow (20 mL min^−1^) of water to the chambers from the surrounding water bath. A digital relay timer (SuperPro Hydroponics Recycling Timer, Xiamen, China) was used to stop water flow for five minutes and then resume flushing for 10 minutes, continuously, for the duration of the trial. Water flow was stopped for five minutes to ensure that O_2_ did not fall below 80% saturation. Fish remained in chambers for four hours to recover to *Ṁ*O_2Rest_. While adult fish typically remain in chambers for up to 24 hours^84^, smaller fish recover much more quickly from exhaustive exercise and are commonly measured for only 2–3 hours to minimize stress and the risk of starvation^85–89^. The temperature-compensated oxygen concentration (mg L^−1^) of the water in each chamber was continuously recorded every 2 seconds (0.5 Hz) using oxygen-sensitive REDFLASH dye on contactless spots (2 mm) adhered to a glass tube in line with the chamber, and linked to a Firesting Optical Oxygen Meter (Pyro Science e. K., Aachen, Germany) with 2 m fibre-optic cables.

Oxygen uptake rates were calculated using linear least squares regression using LabChart version 7.2.5 (ADInstruments, Colorado Springs, CO, USA). Background microbial respiration was subtracted from total chamber respiration to determine the oxygen uptake rate of the fish, as per Rummer *et al*.^81^. The value of *Ṁ*O_2Max_ was determined to be the maximum slope (30 second intervals) immediately following the exhaustive chase. The value of *Ṁ*O_2Rest_ was calculated as the average of the lowest 10% of slopes during the trial, excluding outliers above or below 2 SD. Aerobic scope was calculated as the difference between *Ṁ*O_2Max_ and *Ṁ*O_2Rest_. At the end of each trial, fish were euthanized using an overdose of clove oil. Any excess water was removed by blotting with a paper towel, and the fish’s mass (0.5783 ± 0.1433 g; mean ± SD) and standard length (26.5 ± 0.3 mm; mean ± SD) were recorded. Between trials, the water bath, chambers, and pumps were cleaned with a 10% bleach solution and freshwater to minimize bacterial growth. Between 6 and 14 individuals from each of four family groups were tested per treatment, for a grand total of 37–38 fish per treatment.

### Statistical Analyses

Linear mixed-effects models (LME, “nlme” package in R) were used to determine the effect of CO_2_ and temperature on behavioural and physiological traits. For percent reduction in feeding strikes, the CO_2_ and temperature treatments were fixed effects, with fish mass and time of day as covariates, allowing for interactions between CO_2_ treatment, temperature treatment, mass, and time of day. Family was included as a random factor to account for the possibility that sibling responses were more similar to each other than non-sibling responses. Mass and time of day were mean-centred to help with the interpretation of model intercepts. For physiological traits (aerobic scope, *Ṁ*O_2Rest_, and *Ṁ*O_2Max_), CO_2_ and temperature treatments and mean-centred mass were fixed effects, and family was a random effect. Assumptions of normality and homogeneity of residuals were visually assessed with Q-Q plots and frequency distributions. When the variance of the model residuals increased as the fitted values increased, a power variance function was used to reduce heteroscedasticity. Parameters were estimated using restricted maximum-likelihood. Covariates and interactions between the fixed factor and covariates were dropped when not significant for model simplification and fit. P-values were calculated from the “nlme” package in R, and results were considered statistically significant at P < 0.05. *Post hoc* multiple comparisons were done using the R function “glht” in the package “multcomp” using Tukey’s HSD contrasts for unequal sample sizes.

The relationship between behavioural and physiological performance was modelled using a linear mixed effects model for each treatment. Here we were interested in describing the relationship between traits, and not necessarily assigning causation. We used linear mixed effect models in order to incorporate additional random effects. For each linear mixed effect model, aerobic scope was the dependent variable and percent reduction in feeding strikes was the independent variable. Mean-centred mass was included as a covariate, and family was included as a random factor. When there was a significant relationship observed, linear regressions were used to determine if there were differences between families, with aerobic scope as the dependent variable and percent reduction in feeding strikes, mass, and family as covariates. All analyses were conducted using R version 3.1.3^90^.

## Data Availability

The datasets generated and analysed during the current study are available from the corresponding author on request or via the Tropical Research Data Hub (doi: 10.25903/5ba8882d93620).
